# Supplementary material for: Cis and trans regulatory mechanisms of extrachromosomal DNA segregation
Source: Nat Cell Biol. 2026 Jun 24;28(7):1453–63. doi: 10.1038/s41556-026-01982-0 (PMC13364683; doi:10.1038/s41556-026-01982-0)
Supplement: Supplementary file 1 — Reporting Summary [file 41556_2026_1982_MOESM1_ESM.pdf]

nature portfolio

Corresponding author(s): Sihan Wu

Last updated by author(s): Apr 15, 2026

Reporting Summary

Nature Portfolio wishes to improve the reproducibility of the work that we publish. This form provides structure for consistency and transparency in reporting. For further information on Nature Portfolio policies, see our Editorial Policies and the Editorial Policy Checklist.

Statistics

For all statistical analyses, confirm that the following items are present in the figure legend, table legend, main text, or Methods section.

n/a	Confirmed	
☐	☒	The exact sample size (*n*) for each experimental group/condition, given as a discrete number and unit of measurement
☐	☒	A statement on whether measurements were taken from distinct samples or whether the same sample was measured repeatedly
☐	☒	The statistical test(s) used AND whether they are one- or two-sided<br>*Only common tests should be described solely by name; describe more complex techniques in the Methods section.*
☒	☐	A description of all covariates tested
☐	☒	A description of any assumptions or corrections, such as tests of normality and adjustment for multiple comparisons
☐	☒	A full description of the statistical parameters including central tendency (e.g. means) or other basic estimates (e.g. regression coefficient) AND variation (e.g. standard deviation) or associated estimates of uncertainty (e.g. confidence intervals)
☐	☒	For null hypothesis testing, the test statistic (e.g. *F*, *t*, *r*) with confidence intervals, effect sizes, degrees of freedom and *P* value noted<br>*Give P values as exact values whenever suitable.*
☒	☐	For Bayesian analysis, information on the choice of priors and Markov chain Monte Carlo settings
☒	☐	For hierarchical and complex designs, identification of the appropriate level for tests and full reporting of outcomes
☒	☐	Estimates of effect sizes (e.g. Cohen's *d*, Pearson's *r*), indicating how they were calculated

Our web collection on statistics for biologists contains articles on many of the points above.

Software and code

Policy information about availability of computer code

Data collection	Images were captured and analyzed by Zeiss Axio Observer 7 microscope equipped with the Apotome 3 optical sectioning module, ZEN blue (v3.4, Zeiss). Live-cell imaging was performed with the Zeiss LSM 980 microscope. Western blots were collected with ImageQuant 800 (Cytiva Amersham). qPCR was run on CFX Opus 96 System (Bio-Rad). Infinite M Plex plate reader was used to measure CCK-8 absorbance.
Data analysis	Data were analyzed by R program (version 4.3.2). The fluorescence pixel intensity was measured by ImageJ (1.54g). Western blots were analyzed with and analyzed with Image Lab software (version 6.1.0). qPCR was analyzed with Bio-Rad CFX Maestro 2.3 software (version 5.3.022.1030). Flow cytometry data is analyzed with Kaluza Analysis software (v2.1, Beckman Coulter).<br>For HiC data analysis: Adapter sequences were trimmed by the fastp software (v0.22.0). Trimmed reads were then processed through the HiC-Pro pipeline (v3.1.0) with hg38 as the reference genome. The reproducibility of HiC libraries was analysed by the HiCRep software (v0.2.6). HiC interaction was detected by the FitHiC2 software (v2.0.8).<br>For the ChIP-seq signal on ecDNA: ecDNA signals were normalised with copy number by the bigwigCompare function (--operation ratio) from deepTools (v3.5.5) software, with the ChIP-seq input serving as a surrogate for whole-genome coverage.

For manuscripts utilizing custom algorithms or software that are central to the research but not yet described in published literature, software must be made available to editors and reviewers. We strongly encourage code deposition in a community repository (e.g. GitHub). See the Nature Portfolio guidelines for submitting code & software for further information.

Data

Policy information about availability of data

All manuscripts must include a data availability statement. This statement should provide the following information, where applicable:

- Accession codes, unique identifiers, or web links for publicly available datasets
- A description of any restrictions on data availability
- For clinical datasets or third party data, please ensure that the statement adheres to our policy

All sequencing data are available in SRA database under BioProject accession PRJNA1263546.

Research involving human participants, their data, or biological material

Policy information about studies with human participants or human data. See also policy information about sex, gender (identity/presentation), and sexual orientation and race, ethnicity and racism.

Reporting on sex and gender	Not applicable.
Reporting on race, ethnicity, or other socially relevant groupings	Not applicable.
Population characteristics	Not applicable.
Recruitment	Not applicable.
Ethics oversight	Not applicable.

Note that full information on the approval of the study protocol must also be provided in the manuscript.

Field-specific reporting

Please select the one below that is the best fit for your research. If you are not sure, read the appropriate sections before making your selection.

☒ Life sciences ☐ Behavioural & social sciences ☐ Ecological, evolutionary & environmental sciences

For a reference copy of the document with all sections, see nature.com/documents/nr-reporting-summary-flat.pdf

Life sciences study design

All studies must disclose on these points even when the disclosure is negative.

Sample size	Three biological replicates were done independently for the imaging experiments with total >90 cells were pooled from biological replicates for quantification, unless specified. For the sequencing experiments, two biological replicates were done. For the rest of the experiments, at least three biological replicates were done.
Data exclusions	No data we generated were excluded.
Replication	Three biological replicates were done independently for the experiments, unless specified. For the sequencing experiments, two biological replicates were done.
Randomization	The present study uses cultured human cancer cells for experimental intervention, and randomization is not applicable in this case.
Blinding	All experimental data were acquired by instrumental analysis objectively without anthropic bias, and blinding is not relevant to this study.

Reporting for specific materials, systems and methods

We require information from authors about some types of materials, experimental systems and methods used in many studies. Here, indicate whether each material, system or method listed is relevant to your study. If you are not sure if a list item applies to your research, read the appropriate section before selecting a response.

Materials & experimental systems

n/a	Involved in the study
☐	☒ Antibodies
☐	☒ Eukaryotic cell lines
☒	☐ Palaeontology and archaeology
☒	☐ Animals and other organisms
☒	☐ Clinical data
☒	☐ Dual use research of concern
☒	☐ Plants

Methods

n/a	Involved in the study
☐	☒ ChIP-seq
☐	☒ Flow cytometry
☒	☐ MRI-based neuroimaging

Antibodies

Antibodies used

Flow Cytometry:
anti-phospho-Ser/Thr-Pro MPM-2: Sigma-Aldrich, Cat# 05-368, Lot# 4131152,clone: MPM-2, dilution 1:1000
Goat anti-Mouse IgG (H+L) Highly Cross-Adsorbed Secondary Antibody, Alexa Fluor™ Plus 647: Invitrogen, Cat# A32728, Lot #XH350186, dilution 1:200
F(ab)-Goat anti-Mouse IgG1 Fc Secondary Antibody, Alexa Fluor™ 647: Invitrogen, Cat#A66784, Lot# 2945322, dilution 1:200

Mitotic phase and interphase low-input ChIP-seq:
anti-H3K27ac: EpiCypher, Cat# 13-0059, Lot# 24008001-81,clone: 2114-3E4, 0.5 µg per ChIP
anti-H3K4me3: Diagenode, Cat# C15410003, Lot# A1051D,clone: polyclonal, 0.5 µg per ChIP
anti-BRD4: Bethyl Laboratories, Cat# A301-985A, Lot# 8,clone: polyclonal, 0.5 µg per ChIP
anti-SMARCA4: CST, Cat# 49360S, Lot# 3, clone: D1Q7F, 0.5 µg per ChIP
anti-p-Pol II Ser2/5: CST, Cat# 13546S, Lot# 1, clone: D1G3K, 0.5 µg per ChIP
anti-Pol II: CST, Cat# 2629S, Lot# 4, clone: 4H8, 0.5 µg per ChIP
anti-MED14: Abcam, Cat# ab72141, Lot# 1085218-2, clone: polyclonal, 0.5 µg per ChIP
anti-H3S10phS28ph: Active Motif, Cat# 39148, Lot#140, clone: polyclonal, 0.5 µg per ChIP

Interphase ChIP-seq:
anti-BRD4: Bethyl Laboratories, Cat# A301-985A, Lot# 8, clone: polyclonal, 5 µg per ChIP
anti-SMARCA4: Abcam, Cat# 110641, Lot# 1095241-25,clone: EPNCIR111A 5 µg per ChIP
Spike-in antibody: Active Motif, Cat# 61686, Lot# 24347130-11, 2 µg per ChIP

Western Blot:
anti-H3K27ac: CST, Cat# 8173S, Lot# 8,clone: D5E4, dilution 1:1000
anti-H3K4me3: CST, Cat# 9751S, Lot# 16,clone: C42D8, dilution 1:1000
anti-GAPDH: Proteintech, Cat# 60004-1-Ig, Lot# 10028230, clone: 1E6D9, dilution 1:10000
anti-BRD2: Bethyl Laboratories, Cat# A302-583A, Lot# 7, clone: polyclonal, dilution 1:1000
anti-BRD3: Bethyl Laboratories, Cat# A700-069, Lot# 2, clone: BLR069G, dilution 1:1000
anti-BRD4: Bethyl Laboratories, Cat# A301-985A, Lot# 8, clone: polyclonal, dilution 1:1000
anti-BRD4: Ceii Signaling technology, Cat# 13440S,clone: E2A7X, Lot# 10, dilution 1:1000
anti-SMARCA4: CST, Cat# 49360S, Lot# 3, clone: D1Q7F, dilution 1:1000
anti-p-Pol II Ser2/5: CST, Cat# 13546S, Lot# 1,clone: D1G3K, dilution 1:1000
anti-Pol II: CST, Cat# 2629S, Lot# 4,clone: 4H8, dilution 1:1000
anti-MYC: Abcam Cat# ab32072, Lot# 1053331-31, clone: Y69 dilution 1:2000
anti-DHFR: CST Cat# 43497S, Lot# 1,clone: E6L1H, dilution 1:1000
anti-CDK9: CST Cat# 2316S, Lot# 10, clone: C12F7, dilution 1:1000
anti-Cas9: Sigma-Aldrich, Cat# SAB4200701, Lot# 0000214445,clone: 7A9-3A3, dilution 1:1000
anti-Vinculin: Proteintech Cat# 66305-1-Ig, Lot# 10020241, clone: 2B5A7 dilution 1:10000
secondary anti-rabbit IgG antibody: CST, Cat# 7074S, Lot# 33, dilution 1:5000
secondary anti-mouse IgG antibody: CST, Cat# 7076S, Lot# 39, dilution 1:5000

CUT&RUN:
anti-IgG: EpiCypher, Cat# 13-0042, Lot# 23212006-14, clone: polyclonal, 0.5 µg per reaction
anti-H3K27ac: EpiCypher, Cat# 13-0059, Lot# 24008001-81, clone: 2114-3E4, 0.5 µg per reaction

Immunofluorescence:
anti-H3K27ac: CST, Cat# 8173S, Lot# 8, clone: D5E4, dilution 1:50/1:100
anti-H3K4me3: CST, Cat# 9751S, Lot# 16, clone: C42D8, dilution 1:100
anti-H3S10phS28ph: Active Motif, Cat# 39148, Lot#140, clone: polyclonal, dilution 1:400
Goat anti-Rabbit IgG (H+L) Highly Cross-Adsorbed Secondary Antibody, Alexa Fluor™ Plus 488: Invitrogen, Cat#A32731, Lot#WI329965, dilution 1:200
Goat anti-Rabbit IgG (H+L) Highly Cross-Adsorbed Secondary Antibody, Alexa Fluor™ Plus 594:Invitrogen, Cat#A32740, Lot#WK333741, dilution 1:200

Validation

All antibodies are validated to react with corresponding human antigens. Citation data are acquired from CiteAb database or their manufacturer websites.
Flow Cytometry:
anti-phospho-Ser/Thr-Pro MPM-2: Sigma-Aldrich, Cat# 05-368, 242 citations

Goat anti-Mouse IgG (H+L) Highly Cross-Adsorbed Secondary Antibody, Alexa Fluor™ Plus 647: Invitrogen, Cat# A32728, 760 citations
F(ab)-Goat anti-Mouse IgG1 Fc Secondary Antibody, Alexa Fluor™ 647: Invitrogen, Cat#A66784, used by PMID: 39000003

Mitotic phase and interphase low-input ChIP-seq:
anti-H3K27ac: EpiCypher, Cat# 13-0059, used by PMID: 40324882
anti-H3K4me3: Diagenode, Cat# C15410003, used by PMID: 39797762
anti-BRD4: Bethyl Laboratories, Cat# A301-985A, used by PMID: 39251822
anti-SMARCA4: CST, Cat# 49360S, 79 citations
anti-p-Pol II Ser2/5: CST, Cat# 13546S, 12 citations
anti-Pol II: CST, Cat# 2629S, 143 citations
anti-MED14: Abcam, Cat# ab72141, 3 citations
anti-H3S10phS28ph: Active Motif, Cat# 39148, 1 citation

Interphase ChIP-seq:
anti-BRD4: Bethyl Laboratories, Cat# A301-985A, used by PMID: 39251822
anti-SMARCA4: Abcam, Cat# 110641, 257 citations
Spike-in antibody: Active Motif, Cat# 61686, used by PMID: 33154377

Western Blot:
anti-H3K27ac: CST, Cat# 8173S, 585 citations
anti-H3K4me3: CST, Cat# 9751S, 702 citations
anti-GAPDH: Proteintech, Cat# 60004-1-Ig, 12263 citations
anti-BRD2: Bethyl Laboratories, Cat# A302-583A, used by PMID: 39251822
anti-BRD3: Bethyl Laboratories, Cat# A700-069, used by PMID: 39251822
anti-BRD4: Bethyl Laboratories, Cat# A301-985A, used by PMID: 39251822
anti-BRD4: Ceii Signaling technology, Cat# 13440S, 217 citations
anti-SMARCA4: CST, Cat# 49360S, 79 citations
anti-p-Pol II Ser2/5: CST, Cat# 13546S, 12 citations
anti-Pol II: CST, Cat# 2629S, 143 citations
anti-MYC: Abcam Cat# ab32072, 1721 citations
anti-DHFR: CST Cat# 43497S, 2 citations
anti-CDK9: CST Cat# 2316S, 146 citations
anti-Cas9: Sigma-Aldrich, Cat# SAB4200701, 13 citations
anti-Vinculin: Proteintech Cat# 66305-1-Ig, 199 citations
secondary anti-rabbit IgG antibody: CST, Cat# 7074S, 18146 citations
secondary anti-mouse IgG antibody: CST, Cat# 7076S, 10945 citations

CUT&RUN:
anti-IgG: EpiCypher, Cat# 13-0042, used by PMID: 39729987
anti-H3K27ac: EpiCypher, Cat# 13-0059, used by PMID: 40324882

Immunofluorescence:
anti-H3K27ac: CST, Cat# 8173S, 585 citations
anti-H3K4me3: CST, Cat# 9751S, 702 citations
anti-H3S10phS28ph: Active Motif, Cat# 39148, 1 citation
Goat anti-Rabbit IgG (H+L) Highly Cross-Adsorbed Secondary Antibody, Alexa Fluor™ Plus 488: Invitrogen, Cat#A32731, 1724 citations
Goat anti-Rabbit IgG (H+L) Highly Cross-Adsorbed Secondary Antibody, Alexa Fluor™ Plus 594:Invitrogen, Cat#A32740, 488 citations

Eukaryotic cell lines

Policy information about cell lines and Sex and Gender in Research

Cell line source(s)

COLO320DM, SNU16, MSTO211H, and DLD1 cells were purchased from ATCC. HeLa parental and HeLa-ecDHFR cell lines were a gift from Dr.Roel Verhaak in Yale University. COLO320DM TetO-MYC TetR-mNeonGreen cell line was a gift from Dr. Paul Mischel in Stanford University.

Authentication

Most cell lines were obtained from ATCC and therefore were authenticated by the vendor. HeLa, HeLa-ecDHFR, and COLO320DM TetO-MYC TetR-mNeonGreen cell lines were authenticated by the original labs.

Mycoplasma contamination

All cell-lines were tested negative for mycoplasma.

Commonly misidentified lines
(See ICLAC register)

None of the cell line is listed in ICLAC Register of Misidentified Cell Lines.

Plants

Seed stocks

Report on the source of all seed stocks or other plant material used. If applicable, state the seed stock centre and catalogue number. If plant specimens were collected from the field, describe the collection location, date and sampling procedures.

Novel plant genotypes

Describe the methods by which all novel plant genotypes were produced. This includes those generated by transgenic approaches, gene editing, chemical/radiation-based mutagenesis and hybridization. For transgenic lines, describe the transformation method, the number of independent lines analyzed and the generation upon which experiments were performed. For gene-edited lines, describe the editor used, the endogenous sequence targeted for editing, the targeting guide RNA sequence (if applicable) and how the editor was applied.

Authentication

Describe any authentication procedures for each seed stock used or novel genotype generated. Describe any experiments used to assess the effect of a mutation and, where applicable, how potential secondary effects (e.g. second site T-DNA insertions, mosiacism, off-target gene editing) were examined.

ChIP-seq

Data deposition

☒ Confirm that both raw and final processed data have been deposited in a public database such as GEO.

☒ Confirm that you have deposited or provided access to graph files (e.g. BED files) for the called peaks.

Data access links
May remain private before publication.

https://www.ncbi.nlm.nih.gov/bioproject/PRJNA1263546

Files in database submission

PolII_S2_5_mitosis_rep1_r1.fastq.gz;PolII_S2_5_mitosis_rep1_r2.fastq.gz
PolII_S2_5_mitosis_rep2_r1.fastq.gz;PolII_S2_5_mitosis_rep2_r2.fastq.gz
SMARCA4_mitosis_rep1_r1.fastq.gz;SMARCA4_mitosis_rep1_r2.fastq.gz
SMARCA4_mitosis_rep2_r1.fastq.gz;SMARCA4_mitosis_rep2_r2.fastq.gz
BRD4_mitosis_rep1_r1.fastq.gz;BRD4_mitosis_rep1_r2.fastq.gz
BRD4_mitosis_rep2_r1.fastq.gz;BRD4_mitosis_rep2_r2.fastq.gz
H3K27ac_mitosis_rep1_r1.fastq.gz;H3K27ac_mitosis_rep1_r2.fastq.gz
H3K27ac_mitosis_rep2_r1.fastq.gz;H3K27ac_mitosis_rep2_r2.fastq.gz
PolII_mitosis_rep1_r1.fastq.gz;PolII_mitosis_rep1_r2.fastq.gz
PolII_mitosis_rep2_r1.fastq.gz;PolII_mitosis_rep2_r2.fastq.gz
H3K4me3_mitosis_rep2_r1.fastq.gz;H3K4me3_mitosis_rep2_r2.fastq.gz
INPUT_mitosis_rep1_r1.fastq.gz;INPUT_mitosis_rep1_r2.fastq.gz
INPUT_mitosis_rep2_r1.fastq.gz;INPUT_mitosis_rep2_r2.fastq.gz
MED14_mitosis_rep1_r1.fastq.gz;MED14_mitosis_rep1_r2.fastq.gz
MED14_mitosis_rep2_r1.fastq.gz;MED14_mitosis_rep2_r2.fastq.gz
PolII_S2_5_interphase_rep1_r1.fastq.gz;PolII_S2_5_interphase_rep1_r2.fastq.gz
PolII_S2_5_interphase_rep2_r1.fastq.gz;PolII_S2_5_interphase_rep2_r2.fastq.gz
SMARCA4_interphase_rep1_r1.fastq.gz;SMARCA4_interphase_rep1_r2.fastq.gz
SMARCA4_interphase_rep2_r1.fastq.gz;SMARCA4_interphase_rep2_r2.fastq.gz
BRD4_interphase_rep1_r1.fastq.gz;BRD4_interphase_rep1_r2.fastq.gz
BRD4_interphase_rep2_r1.fastq.gz;BRD4_interphase_rep2_r2.fastq.gz
MED14_interphase_rep1_r1.fastq.gz;MED14_interphase_rep1_r2.fastq.gz
MED14_interphase_rep2_r1.fastq.gz;MED14_interphase_rep2_r2.fastq.gz
PolII_interphase_rep1_r1.fastq.gz;PolII_interphase_rep1_r2.fastq.gz
PolII_interphase_rep2_r1.fastq.gz;PolII_interphase_rep2_r2.fastq.gz
H3K27ac_interphase_rep1_r1.fastq.gz;H3K27ac_interphase_rep1_r2.fastq.gz
H3K27ac_interphase_rep2_r1.fastq.gz;H3K27ac_interphase_rep2_r2.fastq.gz
INPUT_interphase_rep1_r1.fastq.gz;INPUT_interphase_rep1_r2.fastq.gz
INPUT_interphase_rep2_r1.fastq.gz;INPUT_interphase_rep2_r2.fastq.gz
H3K4me3_interphase_rep1_r1.fastq.gz;H3K4me3_interphase_rep1_r2.fastq.gz
H3K4me3_interphase_rep2_r1.fastq.gz;H3K4me3_interphase_rep2_r2.fastq.gz

AU15330_INPUT_rep2_r1.fastq.gz;AU15330_INPUT_rep2_r2.fastq.gz
AU15330_BRD4_rep1_r1.fastq.gz;AU15330_BRD4_rep1_r2.fastq.gz
AU15330_SMARCA4_rep2_r1.fastq.gz;AU15330_SMARCA4_rep2_r2.fastq.gz
AU15330_SMARCA4_rep1_r1.fastq.gz;AU15330_SMARCA4_rep1_r2.fastq.gz
AU15330_BRD4_rep2_r1.fastq.gz;AU15330_BRD4_rep2_r2.fastq.gz
AU15330_INPUT_rep1_r1.fastq.gz;AU15330_INPUT_rep1_r2.fastq.gz
dBET6_INPUT_rep1_r1.fastq.gz;dBET6_INPUT_rep1_r2.fastq.gz
dBET6_INPUT_rep2_r1.fastq.gz;dBET6_INPUT_rep2_r2.fastq.gz
dBET6_BRD4_rep1_r1.fastq.gz;dBET6_BRD4_rep1_r2.fastq.gz
dBET6_BRD4_rep2_r1.fastq.gz;dBET6_BRD4_rep2_r2.fastq.gz
dBET6_SMARCA4_rep1_r1.fastq.gz;dBET6_SMARCA4_rep1_r2.fastq.gz
dBET6_SMARCA4_rep2_r1.fastq.gz;dBET6_SMARCA4_rep2_r2.fastq.gz
DMSO_SMARCA4_rep1_r1.fastq.gz;DMSO_SMARCA4_rep1_r2.fastq.gz
DMSO_SMARCA4_rep2_r1.fastq.gz;DMSO_SMARCA4_rep2_r2.fastq.gz
DMSO_BRD4_rep2_r1.fastq.gz;DMSO_BRD4_rep2_r2.fastq.gz

DMSO_INPUT_rep1_r1.fastq.gz;DMSO_INPUT_rep1_r2.fastq.gz
DMSO_INPUT_rep2_r1.fastq.gz;DMSO_INPUT_rep2_r2.fastq.gz
DMSO_BRD4_rep1_r1.fastq.gz;DMSO_BRD4_rep1_r2.fastq.gz

M_input_r1.fastq.gz;M_input_r2.fastq.gz
M_S10S28_rep1_r1.fastq.gz;M_S10S28_rep1_r2.fastq.gz
M_S10S28_rep2_r1.fastq.gz;M_S10S28_rep2_r2.fastq.gz
G1_input_r1.fastq.gz;G1_input_r2.fastq.gz
G1_S10S28_rep1_r1.fastq.gz;G1_S10S28_rep1_r2.fastq.gz
G1_S10S28_rep2_r1.fastq.gz;G1_S10S28_rep2_r2.fastq.gz

Genome browser session (e.g. UCSC): No longer applicable

Methodology

Replicates: Two biological replicates for each ChIP-seq reactions were performed with similar results.

Sequencing depth: At least 20 Million paired reads (PE50) were obtained per library. Unique mapping rates were above 80%.

Antibodies: See Method section in the manuscript.

Peak calling parameters: macs3 callpeak -t "$CHIP_BAM" -c "$CONTROL_BAM" -f BAM -g hs -n "$OUTPUT_NAME" -q 0.05

Data quality: We used the default setting of MACS3, i.e. the minimum fold change range we used from MACS3 is the default.

Software: BWA-MEM2 (version 2.2.1) for alignment, sambamba (version 1.0.1) for sorting and removing duplicates, bamCoverage (version 3.5.5) to generate visualization tracks.

Flow Cytometry

Plots

Confirm that:

☒ The axis labels state the marker and fluorochrome used (e.g. CD4-FITC).

☒ The axis scales are clearly visible. Include numbers along axes only for bottom left plot of group (a 'group' is an analysis of identical markers).

☒ All plots are contour plots with outliers or pseudocolor plots.

☒ A numerical value for number of cells or percentage (with statistics) is provided.

Methodology

Sample preparation: For Hi-C sample preparation: Single-cell suspensions were cross-linked with 2% formaldehyde (Sigma-Aldrich) for 10 min, quenched with glycine from the respective kit for 10 min, and permeabilized with 0.1% saponin (Thermo Scientific) in PBS for 10 min. After blocking with 1% FBS + 0.1% saponin for 30 min, the anti-phos-MPM2 (Sigma-Aldrich 05-368, 1:1,000) primary antibody was added to the blocking solution and incubated for 1 hr. Cells were then washed 3× with PBS containing 0.1% saponin before AlexaFluor-conjugated secondary antibodies (Invitrogen) staining for another 1 hr. After another 3x washes, DNA was stained with 1 μg mL-1 DAPI for 5 min. Finally, cells were sorted using the FACSAria Fusion (BD Biosciences) for G1 (2N DNA, MPM2-negative) and M phase (4N DNA, MPM2-positive). All procedures were performed at room temperature (RT). To sort for mitotic ChIP-seq: Cells were fixed with 2 mM disuccinimidyl glutarate for 30 min, and then together with 1% formaldehyde (Sigma-Aldrich) for 8 min, and protease inhibitor was added throughout experiment, and the rest of the procedure was the same.

Instrument: FACSAria Fusion (BD Biosciences).

Software: BD FACSDiva™ Software was used for sorting. Data was analyzed using Kaluza Analysis software (v2.1, Beckman Coulter).

Cell population abundance: About 15%-40% of the cells in the population were sorted. After sorting, the sample were checked using microscope with more than 99% cells were mitotic cells.

Gating strategy: We used FSC-A/SSC-A to locate the major cell population, and DAPI-A/DAPI-H to gate single cell. the mitotic cells were further gated by p-MPM2-A and DAPI-A. Detailed method was described in Zhang et al., Nature, 2019.

☒ Tick this box to confirm that a figure exemplifying the gating strategy is provided in the Supplementary Information.
