# Supplementary material for: Cis and trans regulatory mechanisms of extrachromosomal DNA segregation
Source: Nat Cell Biol. 2026 Jun 24;28(7):1453–63. doi: 10.1038/s41556-026-01982-0 (PMC13364683; doi:10.1038/s41556-026-01982-0)

Fig 3

j

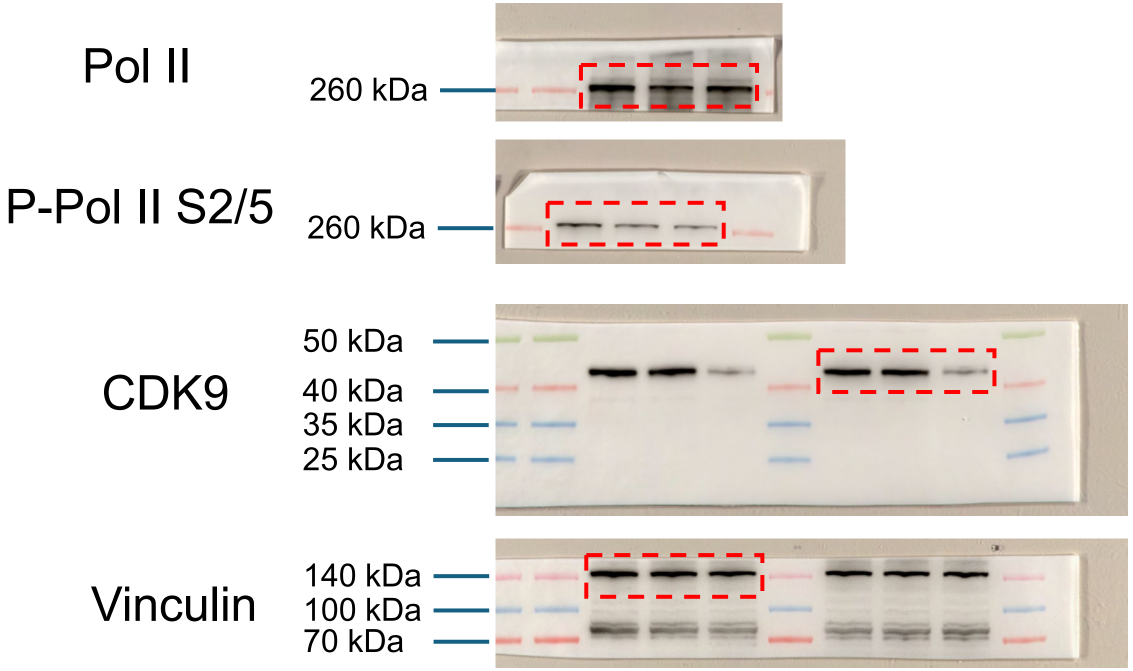
Pol II
260 kDa
P-Pol II S2/5
260 kDa
CDK9
50 kDa
40 kDa
35 kDa
25 kDa
Vinculin
140 kDa
100 kDa
70 kDa

Extended Data Fig 2

g

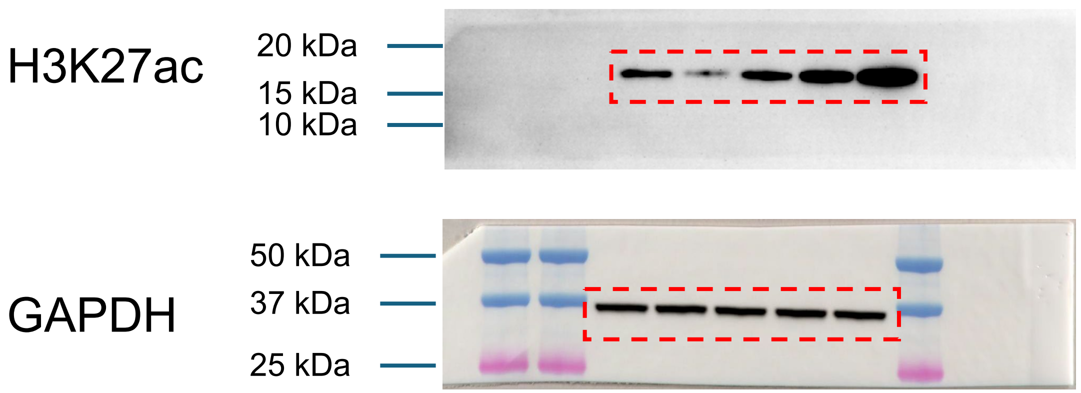

n

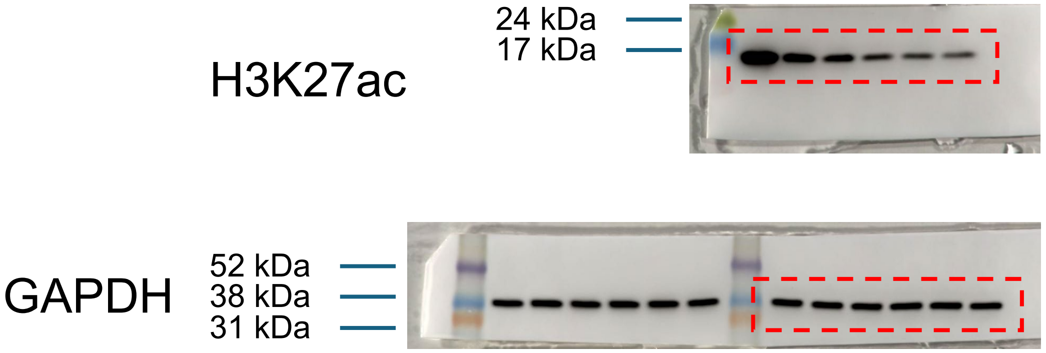

o

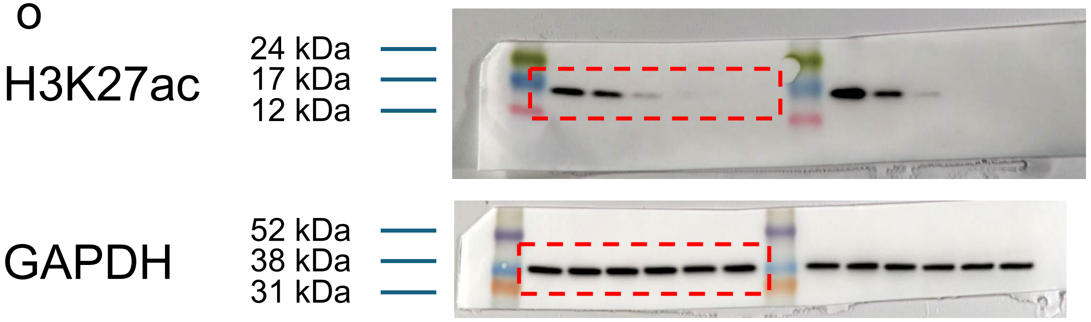

Extended Data Fig 3

a

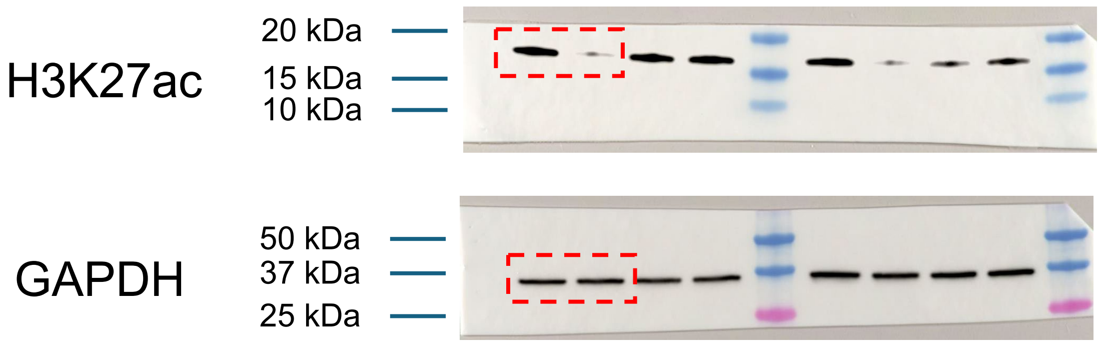

d

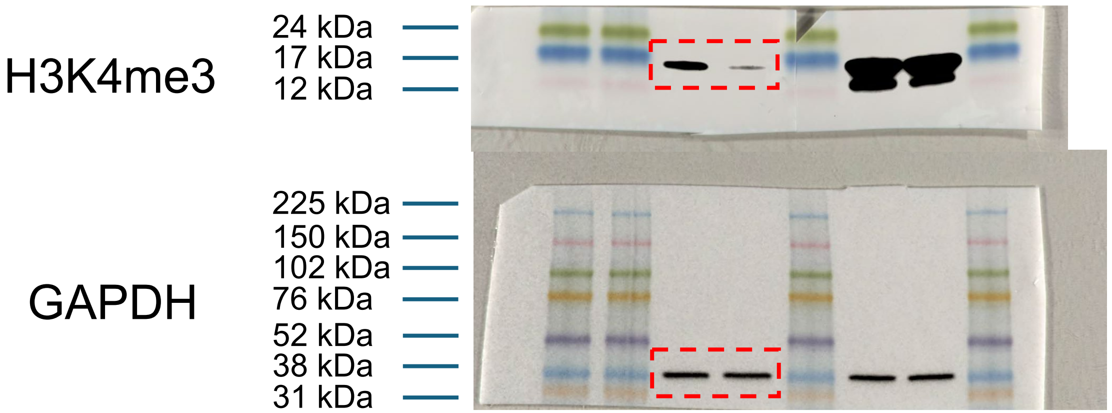

Extended Data Fig 4

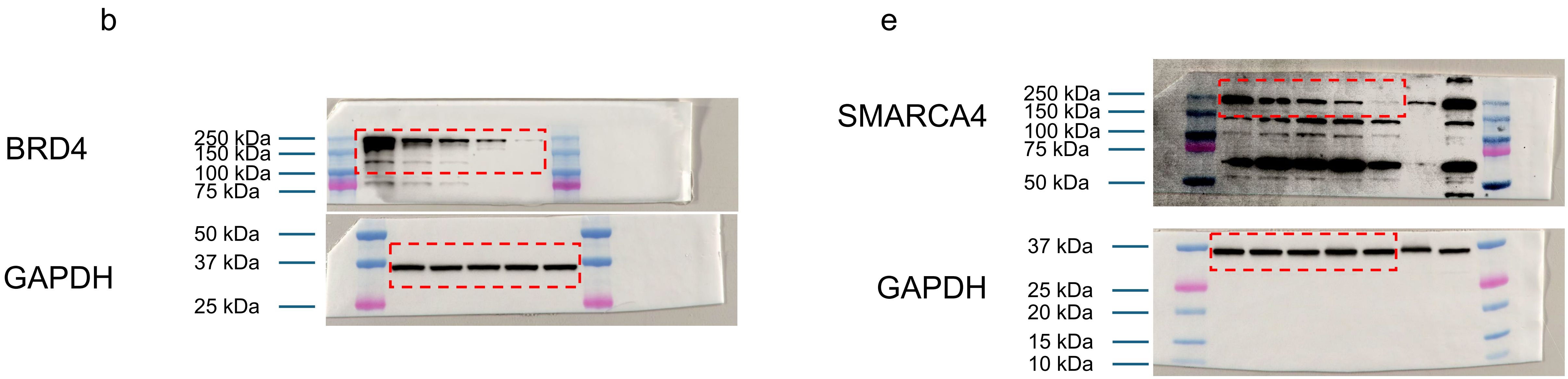

Extended Data Fig 5

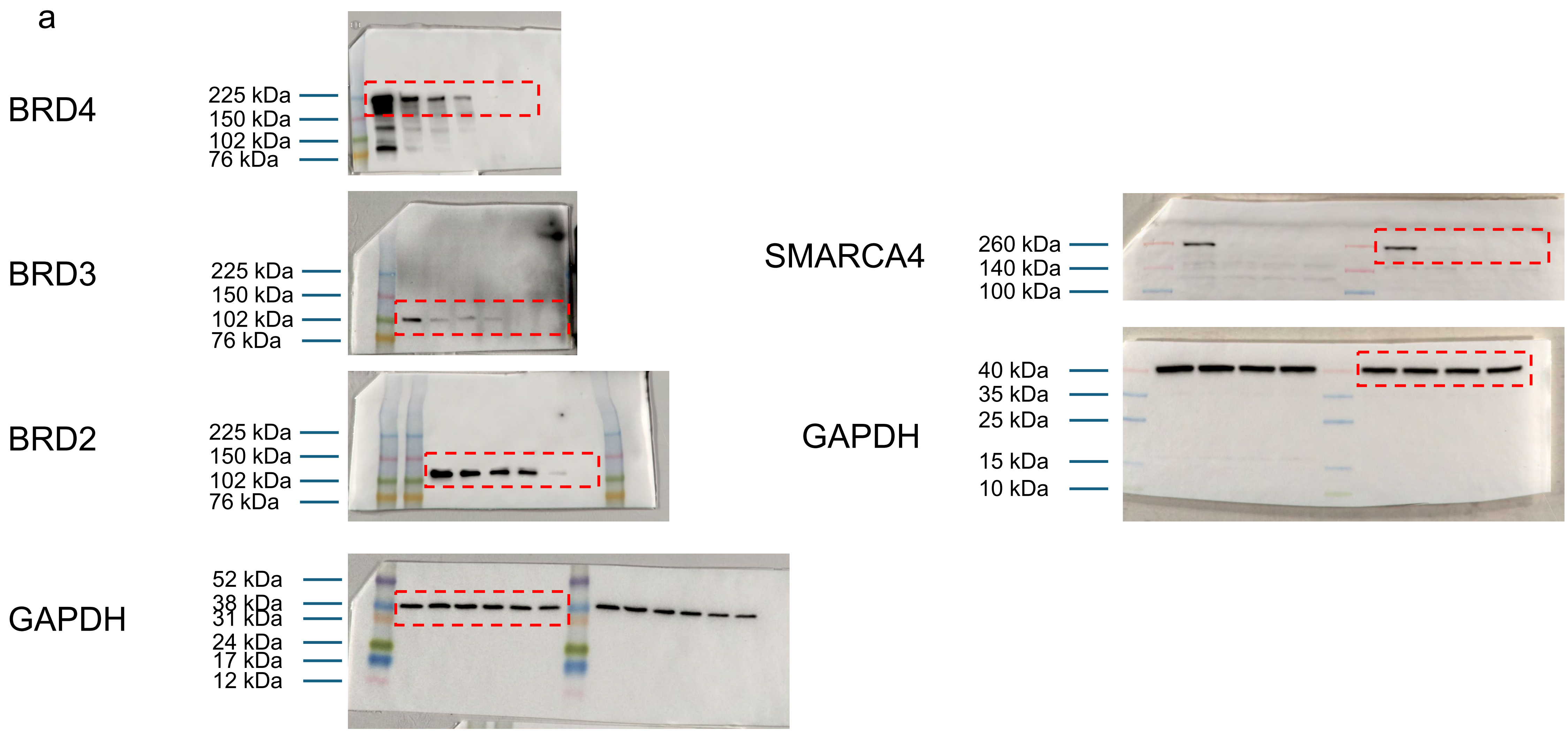

Extended Data Fig 7

b

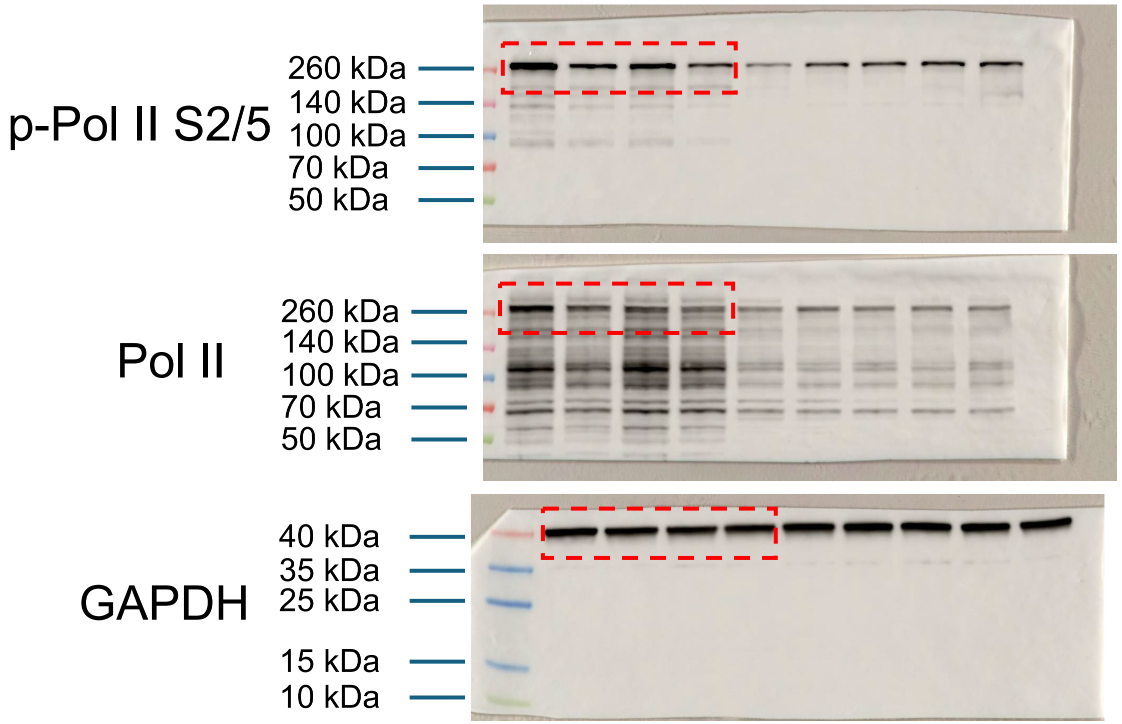

g

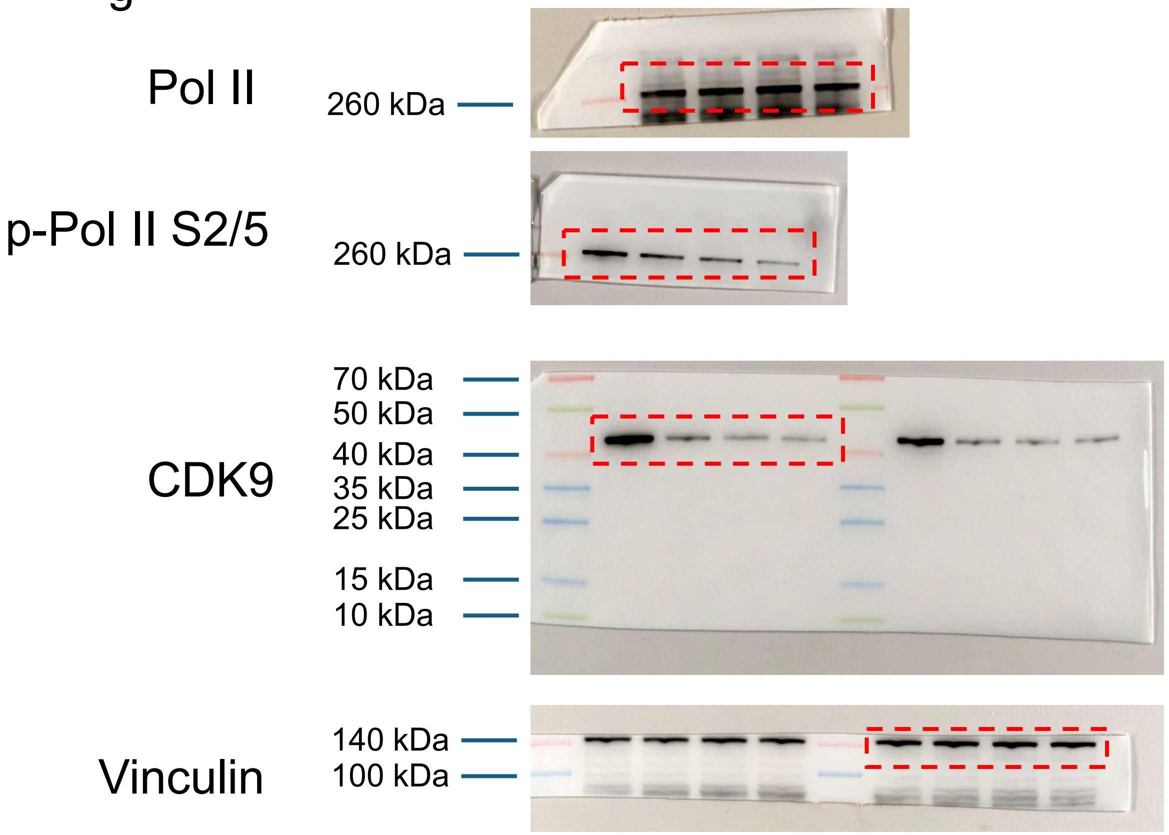

Extended Data Fig 8

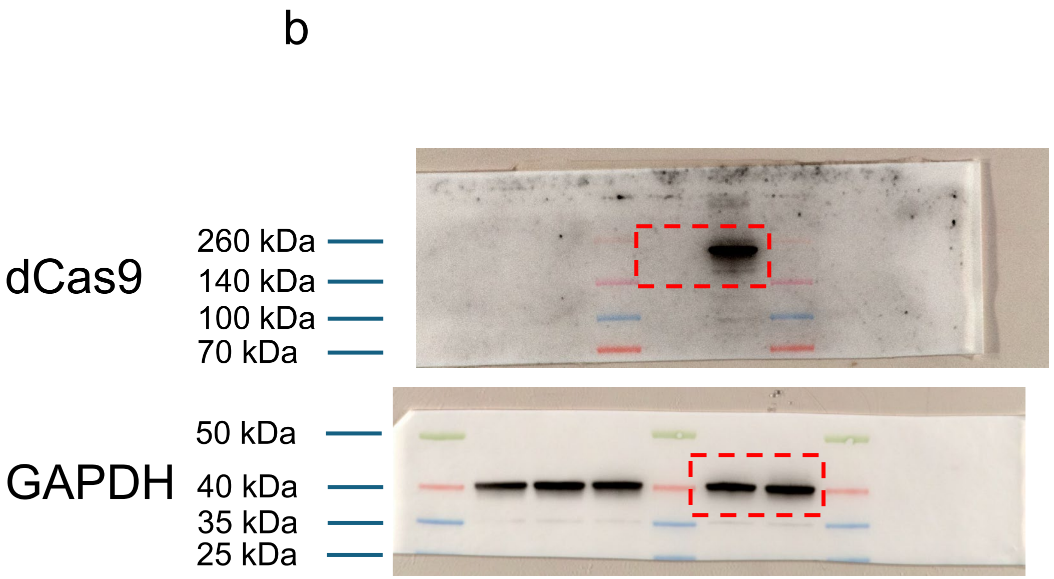

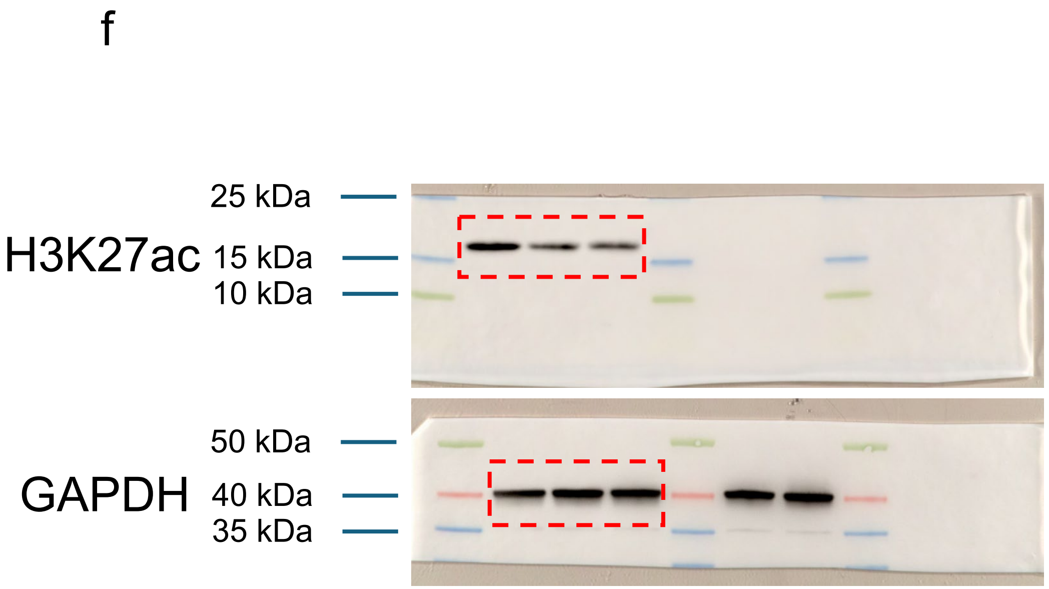

Extended Data Fig 9

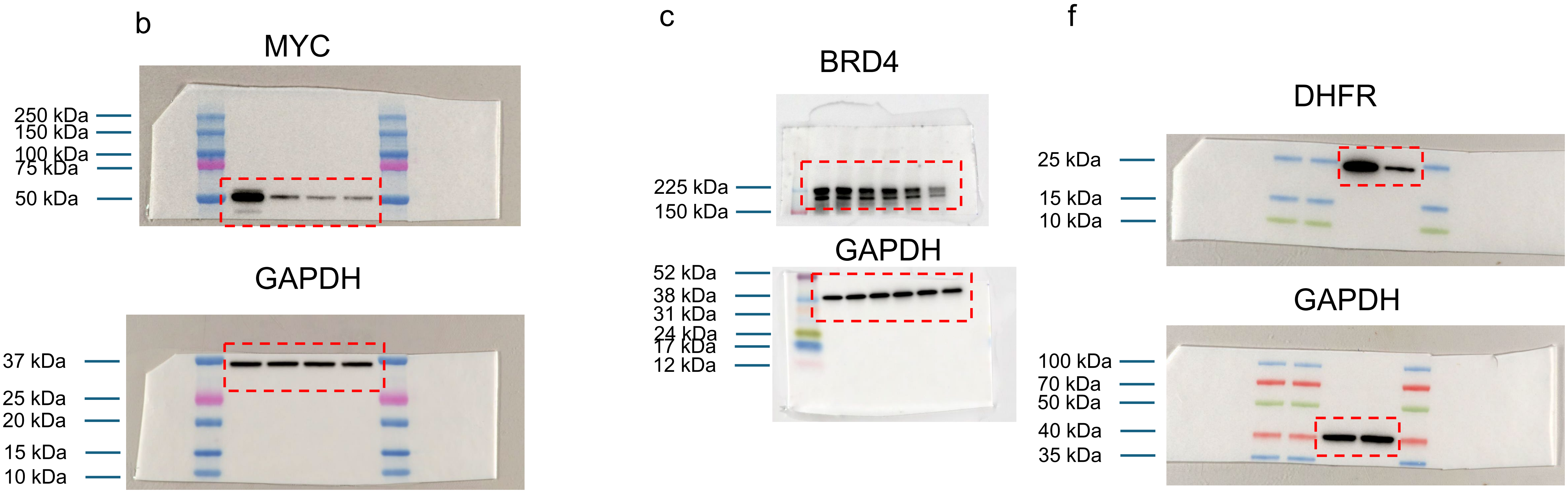

Supplement: Supplementary file 4 — Uncropped western blots. [file 41556_2026_1982_MOESM4_ESM.pdf]
